# Temporal Trends in Transcatheter Aortic Valve Replacement for Isolated Severe Aortic Stenosis

**DOI:** 10.1016/j.jscai.2024.101861

**Published:** 2024-04-05

**Authors:** Tanush Gupta, James T. DeVries, Fahad Gilani, Ansar Hassan, Cathy S. Ross, Harold L. Dauerman

**Affiliations:** a Division of Cardiology, University of Vermont Medical Center, Burlington, Vermont; b Division of Cardiology, Dartmouth-Hitchcock Medical Center, Lebanon, New Hampshire; c Division of Cardiology, Catholic Medical Center, Manchester, New Hampshire; d Department of Cardiac Surgery, Maine Medical Center, Portland, Maine

**Keywords:** aortic stenosis, surgical aortic valve replacement, transcatheter aortic valve replacement

Despite 2020 American College of Cardiology (ACC)/American Heart Association (AHA) guidelines that recommend surgical aortic valve replacement (SAVR) for patients with symptomatic severe aortic stenosis (AS) aged <65 years, we demonstrated dramatic growth in transcatheter aortic valve replacement (TAVR) utilization in younger patients aged <65 years from 2015 to 2021 using the United States nationwide Vizient Clinical Data Base.^1^^,^^2^ More recently, a French national registry described more modest growth of TAVR in young patients with isolated AS with only 11.1% of patients aged <65 years receiving TAVR in 2020.^3^ Whether this difference in temporal trend observations is due to registry characteristics, patient characteristics, different years of inclusion, or true differences in national approaches to AS management is unclear. We hypothesized that TAVR adoption has continued to grow among younger patients with isolated severe AS and sought to confirm our prior Vizient-based findings using a different regional US registry with granular data. Further, we sought to update contemporary temporal trends beyond the years represented in the prior French (2015-2020) and US Vizient (2015-2021) studies.^2^^,^^3^

The Northern New England Cardiovascular Disease Group Consortium (NNECDSG) is a regional collaborative of 4 cardiovascular programs in Northern New England (Maine, New Hampshire, and Vermont). We identified all patients who underwent TAVR or isolated SAVR for severe AS from 2016 to 2022. Exclusion criteria included concomitant procedures, endocarditis, emergent procedures, and prior aortic valve replacement. The details of our study design are similar to our prior NNECDSG analysis on AVR trends from 2016 to 2019.^4^ Temporal trends in TAVR vs SAVR utilization were compared according to the 3 guideline-recommended age strata: <65 years, 65 to 80 years, and >80 years. Multivariable logistic regression analysis was used to identify independent predictors of TAVR in patients aged <65 years (variables included age, sex, body mass index, left ventricular ejection fraction, urgent vs elective clinical status, New York Heart Association class IV status, bicuspid aortic valve anatomy, and relevant comorbidities, ie, chronic kidney disease [CKD], chronic obstructive pulmonary disease, diabetes mellitus, congestive heart failure, home oxygen use, peripheral vascular disease, prior coronary artery bypass grafting (CABG), prior percutaneous coronary intervention, and prior stroke). Univariate predictors (*P* < .10) of TAVR were included in the multivariable model. Each site’s institutional review board approved participation in the NNECDSG registry.

Of 6728 patients (40.2% women) undergoing isolated AVR for AS, 1634 (33.4% women) underwent SAVR and 5094 (42.4% women) underwent TAVR. Patients aged <65 years, 65 to 80 years, and >80 years comprised 11.2%, 47.4%, and 41.4% of the overall study population, respectively. For patients aged >80 years, TAVR remained the dominant modality of treatment throughout the study period, with utilization rates increasing from 86.1% in 2016 to 97.8% in 2022 (P_trend_ < .001). In the 65 to 80 years age group, TAVR utilization increased by 2-fold from 42.1% to 80.6% (P_trend_ < .001) (Figure 1A, B).

**Figure 1:**
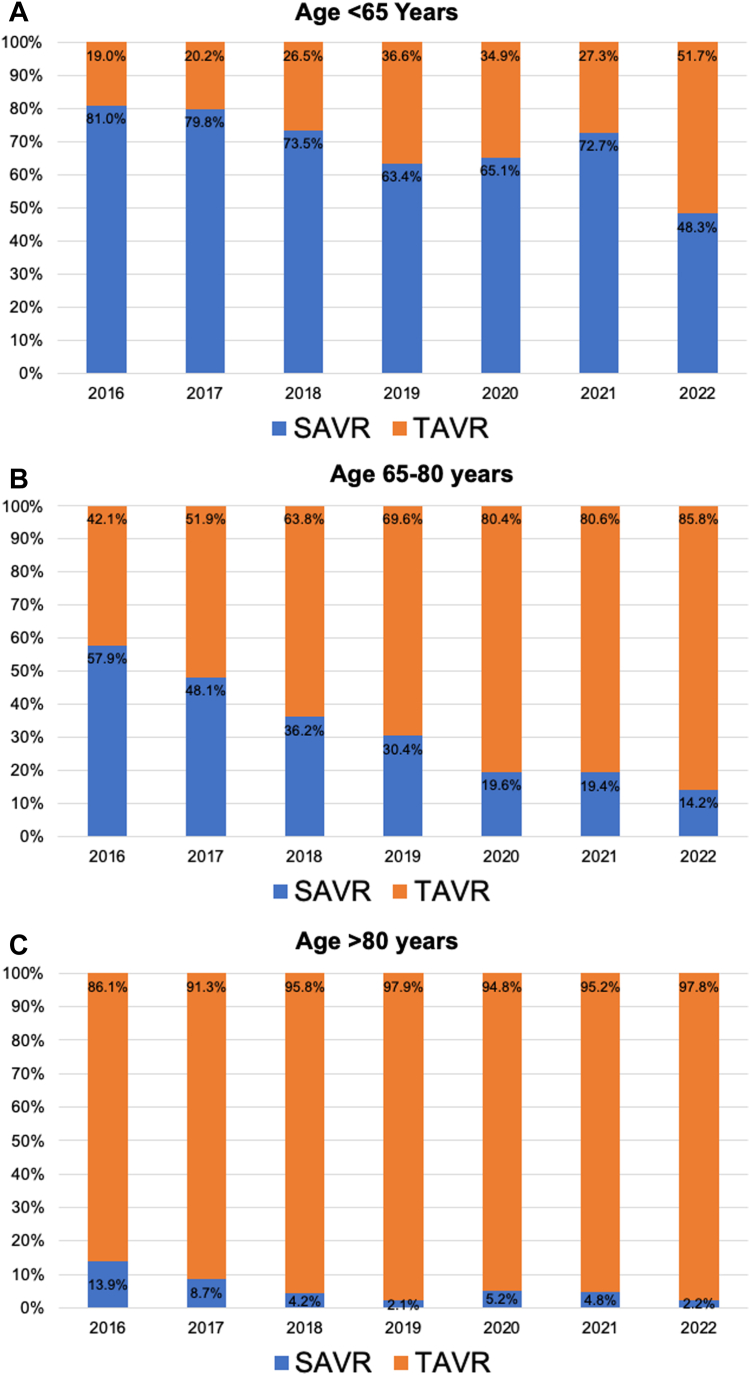
**Trends in utilization of transcatheter aortic valve replacement (TAVR) vs surgical aortic valve replacement (SAVR) by guideline-stratified age groups.** (**A**) Age <65 years; (**B**) age 65 to 80 years; (**C**) age >80 years. The x-axis denotes calendar years; the y-axis denotes percentages. *P* < .001 for all trends.

In patients aged <65 years, TAVR increased dramatically (272%) and by 2022, TAVR comprised 51.7% of all isolated AVRs (Figure 1C). In the <65 years age cohort, the mean age of the patients was 60.7 ± 4.6 years in the TAVR group vs 58.1 ± 6.0 years in the SAVR group (*P* < .001). The average Society of Thoracic Surgeons Predicted Risk of Mortality (STS-PROM) was 3.5 ± 3.1% vs 1.5 ± 1.4% in the TAVR vs SAVR groups, respectively (*P* < .001). Specifically, within the TAVR group, the proportion of patients aged <65 years with low (STS-PROM <4%), intermediate (STS-PROM 4%-8%), and high (STS-PROM ≥8%) surgical risk was 67.4%, 24.7%, and 7.9%, respectively. In the SAVR cohort, 94.6%, 4.6%, and 0.8% had low, intermediate, and high surgical risk, respectively. From 2016 to 2022, the proportion of TAVR patients aged <65 years at low surgical risk, as estimated by STS-PROM, increased from 55% to 73.3%. The observed 30-day mortality rates in patients aged <65 years who underwent TAVR vs SAVR were 0.9% vs 0.8% (*P* = .89).

TAVR patients <65 years old were less likely to have bicuspid aortic valve disease (17.0% vs 44.2%, *P* < .001) and more likely to have prior CABG (17.0% vs 0.8%; *P* < .001). The most powerful independent predictors of TAVR utilization in patients aged <65 years included prior CABG (OR, 33.4; 95% CI, 7.4-152.0), CKD (OR, 3.96; 95% CI, 1.47-10.67), and congestive heart failure (OR, 2.24; 95% CI, 1.39-3.60). On the contrary, bicuspid aortic valve disease was the most potent predictor of SAVR among young patients with isolated severe AS (OR, 0.30; 95% CI, 0.19-.049).

There are 4 main observations from our regional study of AVR temporal trends: (1) these results are concordant with our prior analysis of TAVR utilization in patients aged <65 years in the United States using the Vizient Clinical Database which demonstrated near equalization of TAVR vs SAVR by 2021; (2) to our knowledge, this represents the first temporal trend analysis of TAVR vs SAVR that incorporates both pre-COVID pandemic and the pandemic calendar years; (3) consistent with our 2 prior temporal trends studies, TAVR utilization is predicted by prior CABG and congestive heart failure, while SAVR is predicted by bicuspid aortic valve disease; and (4) we demonstrate that the growth of TAVR in younger adults <65 years of age is driven by increased TAVR utilization in low surgical risk patients.

In contrast, a national registry from France reported that although the proportion of TAVR increased by 63.2% from 2015 to 2020, TAVR comprised only 11.1% of all isolated AVR by 2020.^3^ Given the consistency of our Vizient and Northern New England database analyses, our results are consistent with a true difference in national approaches to the treatment of isolated severe AS between Europe and the US. This hypothesis is also supported by recent data from the California State Discharge Administrative Database that demonstrate 45.7% TAVR utilization in 2021 in young adults aged ≤60 years.^5^ In contrast to the French registry study, we did not use a comorbidity index to analyze predictors of TAVR in younger patients; instead, we analyzed individual univariate clinical predictors and found that TAVR is clearly favored in patients with prior CABG, CKD, and congestive heart failure, while SAVR is favored in young patients with bicuspid aortic valve disease. Notably, our results are consistent using 2 different database analyses.^2^^,^^4^ Finally, the French registry suggested an interaction of sex with TAVR utilization, whereas our study observed a univariate association of TAVR utilization with female sex, and multivariable analysis did not show female sex to be an independent predictor of TAVR utilization in young patients (OR, 1.30, 95% CI, 0.83-2.04). The strength of this study is the use of a unique US multicenter data set and expanded time frame including 2022 data to confirm prior Vizient study results. We do acknowledge that these practice patterns seen in Northern New England might not be reflective of the national US practice. In conclusion, our study suggests that as of 2022, TAVR is now utilized in the majority of adults aged 60 years and older with isolated severe AS with important implications for lifetime management strategies.

## References

[1] Otto C.M., Nishimura R.A., Writing Committee Members (2021). 2020 ACC/AHA guideline for the management of patients with valvular heart disease: a report of the American College of Cardiology/American Heart Association Joint Committee on Clinical Practice Guidelines. J Am Coll Cardiol.

[2] Sharma T., Krishnan A.M., Lahoud R., Polomsky M., Dauerman H.L. (2022). National trends in TAVR and SAVR for patients with severe isolated aortic stenosis. J Am Coll Cardiol.

[3] Prosperi-Porta G., Nguyen V., Willner N. (2023). Association of age and sex with use of transcatheter aortic valve replacement in France. J Am Coll Cardiol.

[4] Sharma T., Tapales A.J.D., Ross C.S. (2022). Concordance of guideline-based risk stratification and selection of patients for transcatheter aortic valve implantation or surgical replacement. Am J Cardiol.

[5] Malas J. Guidelines versus practice: a statewide survival analysis of SAVR versus TAVR in patients aged ≤ 60 Years. https://www.tctmd.com/slide/guidelines-versus-practice-statewide-survival-analysis-savr-versus-tavr-patients-aged-60.

